# Assessment of the impacts of climat change on water supply system pipe failures

**DOI:** 10.1038/s41598-023-33548-7

**Published:** 2023-05-05

**Authors:** Xudong Fan, Xijin Zhang, Allen Yu, Matthew Speitel, Xiong Yu

**Affiliations:** 1 Department of Civil and Environmental Engineering, Case Western Reserve University, 2104 Adelbert Road, Bingham 248, Cleveland, OH 44106-7201 USA; 2 Department of Civil and Environmental Engineering, Case Western Reserve University, 2104 Adelbert Road, Bingham 249C, Cleveland, OH 44106-7201 USA; 3 Beachwood High School, Beachwood, OH 44122 USA; 4 Department of Civil and Environmental Engineering, Case Western Reserve University, 2104 Adelbert Road, Bingham 235, Cleveland, OH 44106-7201 USA; 5 Opal J. and Richard A. Vanderhoof Professor and Chair, Department of Civil and Environmental Engineering, Case Western Reserve University, 2104 Adelbert Road, Bingham 206, Cleveland, OH 44106-7201 USA

**Keywords:** Governance, Civil engineering

## Abstract

Climate change is projected to have profound impacts on the resilience and sustainability of built infrastructure. This study aims to understand the impacts of climate change on water supply systems and to facilitate adaptive actions. A premium database maintained by the Cleveland Water Division, Cleveland, Ohio, USA is analyzed. It contains 29,621 pipe failure records of 51,832 pipes over the past 30 years, representing one of the largest dataset in current literature. From the database, pipe failure rate models have been developed for water pipes made of different types of materials at different ages. The influence of climate (temperature and precipitation) on fragility of water pipes are obtained. Based on the developed climate-fragility failure rate models, the impacts of climate change on the water systems located in different geographic regions are evaluated by predicting the failure rate and number of failures in the water systems in the next 80 years (2020 to 2100). Climate models are used to predict weather under different climate change scenarios. The results demonstrate that the impacts of climate change on water supply system are likely complicated and are dependent upon factors such as the geographic location, pipe material, pipe age, and maintenance strategies. Water pipes in the cold regions may experience fewer number breaks due to the warmer weather and less severe winter, whereas those located in the hot regions may experience more failures associated with more corrosion. Different pipe replacement strategies are compared, which demonstrate the importance of considering the aging of water supply system in future maintenance decisions. This study enriches current understandings on the impacts of climate change on the water systems. The results will help water utilities to design climate change adaptation strategies.

## Introduction

The drinking water distribution system is a backbone infrastructure for modern communities. Failures of water pipes cause expensive direct and indirect costs to the community. Climate is considered one of the significant factors influencing pipe failure^1^. Climate change is expected to alter the temperature and precipitation patterns in the future, which then alters the patterns of pipe failure^2,3^. Additionally, pipes in the current water systems are continuously aging over time, which further exacerbates the failures due to limited budget and underinvestment over the past decades^4^. The condition of drinking water system in the United States has been rated at D by the ASCE Report Card^5^. Therefore, it is crucial to develop water pipe failure models that allow utilities to simultaneously consider the effects of climate change, pipe ages, and pipe materials, etc. Such models will also help utilities to improve long-term planning and to institute policies that lead to more climate-resilient water supply systems^6^.

The temperature and precipitation have been identified as two of the most influential climate factors in water pipe failures^7^. High failure rates were often observed in cold winters. Other factors, such as accidents and natural hazards, are not considered in this study due to the inherent randomness. For the impacts of temperature, previous studies have shown that most water pipe failures occur in the late autumn and winter seasons^8^. Iron pipes have higher failure rates during cold winters, mainly due to temperature-related soil freezing and volume expansion^9^. On the other hand, plastic pipes (PVC or PE) better withstand the effects of thermal expansion and contraction than iron pipes, making them less susceptible to the cold winters^10^. However, plastic pipes were found to be more prone to fail in dry summers^11,12^. Compared to the studies on the influence of temperature, fewer studies have analyzed the impacts of precipitations. Clark^13^ indicated that a long period of a wetting period followed by a long warm and dry period would let to high volume changes of expansive soils, which increased the failure rate of rigid water pipes. Recent studies also demonstrated the close relationship between the precipitation and the pipe failure probabilities^14^.

Different metrics based on climatic temperature or precipitation are often used to capture their correlation with the water pipe failure. Because the water pipes are buried underground, there are time lapses in the demonstration of effects of weather on pipes. For example, study by Laucelli, Rajani^15^ considered the daily average minimum, daily average maximum temperature, variation of temperature, and total rain over a specific period. Wols and van Thienen^11^ considered the rain deficit, mean temperature, and antecedent precipitation index to analyze the climatic effects on pipe failures. Almheiri, Meguid^16^ utilized the freezing and thawing indexes in a vector autoregressive (VAR) model to predict the water system’s failure rates. Rajani, Kleiner^17^ included 11 variables based on the temperature data, including the intensities of temperature change, severity of extreme temperature, and the duration of extreme temperature. Although different metrics based on temperature and precipitation data have been used in previous studies, there is no consensus on the metrics to include on the effects of climate on water pipe failures.

Statistical models have been widely used to study the impacts of climate change on water infrastructure systems due to their simplicity and interpretability^3,15^. It is also suitable for providing a more interpretable results with rich dataset^18^. For example, Żywiec, Boryczko^1^ calculated the failure rate at different temperatures in the study of water system in Poland. Wols, Vogelaar^19^ used the statistical analysis to study the influence of temperature, precipitation, and wind speed on the water system’s failure rate. Although different models have been used to study the impacts of climate factors, only a few studies have used these models to predict the influence of future climate change. To the author’s best knowledge, Wols and Van Thienen^20^ and Żywiec, Piegdoń^21^ are the only studies that have used the statistical relationship between temperature and the water system failure rate to predict the long term water pipe system failure frequency to the end of this century (i.e., from 2010 to 2100). Also, most existing studies did not consider the effects of pipe aging when analyzing the impact of climate changes, probably due to the limitations in the amount of data available to quantify the influence of pipe aging on water pipe failures.

In this work, statistical analyses are performed to develop water pipe failure models for different pipe materials and age groups. The water system under study is managed by the Cleveland Water Division, Cleveland, Ohio, USA, which contains more than 30 years of maintenance records^22^. The historical weather records from the NOAA^23^ are utilized in the analyses. Based on the developed failure models of water pipe fragility over climate, the impacts of climate on the water system are studied by considering different climate zones, climate projected models, and socio-economic paths. The future climate data is collected from the Coupled Model Intercomparison Project Phase 6 (CIMP6)^24^.

## Data and methods

**Figure 1:**
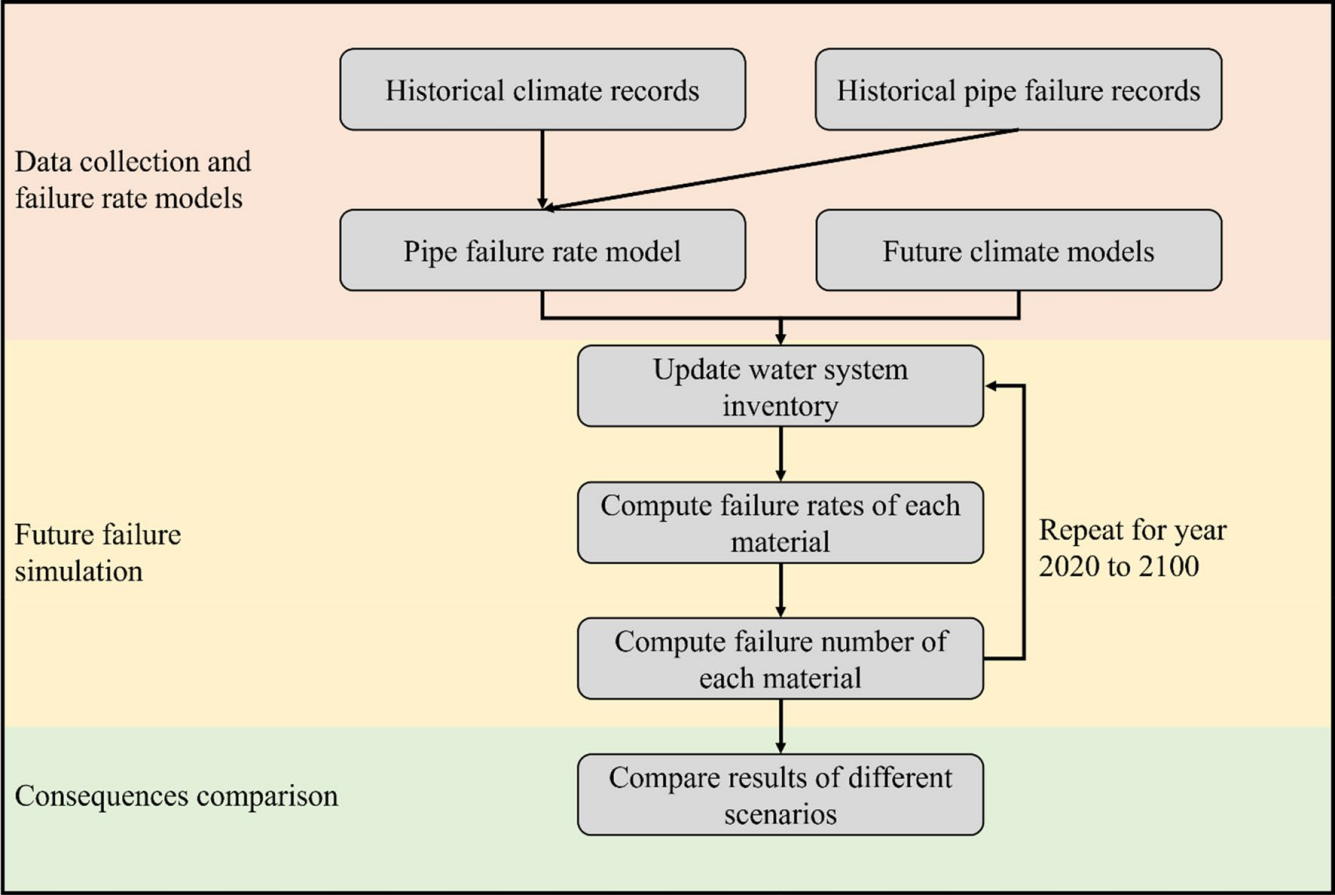
Flow diagram to study the impacts of climate change on the water system.

Figure 1 shows the flowchart used to study the impacts of climate change. The whole diagram includes three major steps, i.e., development of pipe failure rate models for climate fragility, prediction of future failure numbers of the Cleveland water system under different climate change patterns (by assigning it to different geographic regions), and comparison of the consequence of water pipe maintenance schema. "Data sources" and "Pipe failure fragility over climate" sections introduce the data sources and definition of the failure rate. "Simulation and comparison of climate change impacts on water supply system" section elaborates the procedures for future failure prediction and compares the consequence.

### Data sources

Data from different sources are firstly acquired and organized for this study, which includes the following dataset: (1) dataset of the system inventory of the Cleveland water system and historical water pipe failure records^22^, (2) Historical weather data from the related climate stations from the National Oceanic and Atmospheric Administration (NOAA) database^23^, and (3) Future climate data from different models of CMIP6^24^.

**Figure 2:**
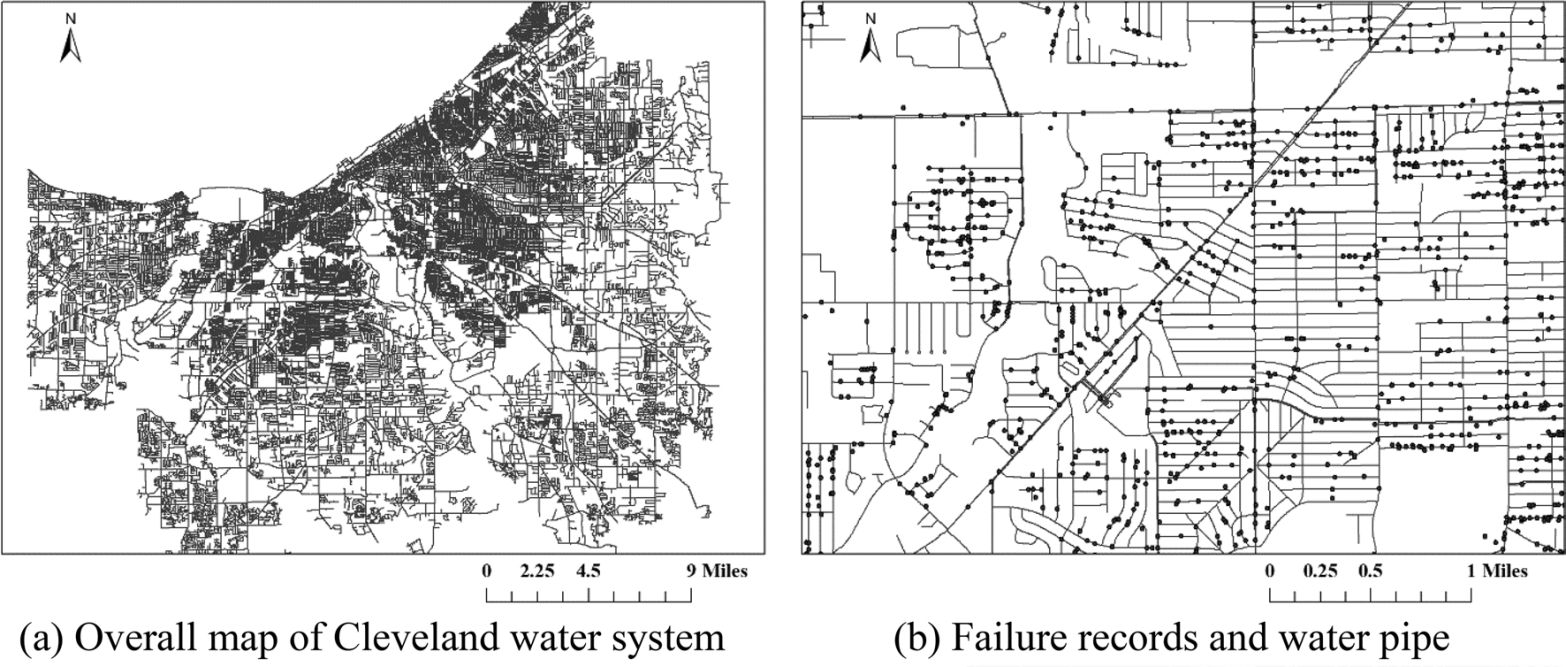
Overview map of the water pipes and failure records (map is generated by CWD data and ArcGIS Pro version 3.1.0 (https://www.esri.com/en-us/arcgis/products/arcgis-pro/overview)).

Figure 2(a) shows the overall map of the assembled dataset, and Fig. 2(b) shows an example of the locations of recorded water failures and water pipes. The physical information of each pipe is stored as ‘Polyline’ in the Geographic Information System (GIS), and the failure information is stored as ‘Point’. Four critical information is extracted from the failure records and inventory dataset, i.e., the (1) pipe material, (2) pipe installation date, (3) pipe length, and (4) failure recorded to date. The temporal resolution of the pipe installation date and pipe failure date is ‘day’, and the unit of pipe length is feet. To reduce the uncertainty, only pipes from three most common classified categories in the database, i.e., the ‘Cast Iron pipes’, ‘Ductile Iron pipes’, and ‘Unknown’, are studied. The total number of these three pipes is 59,303, accounting for 96.01% of the total system. The corresponding number of maintenance records is 28,520, accounting for 96.28% of the total maintenance records. The Cleveland Water Department began to keep the record the pipe failures in 1985. Since there are significant amount of missing records in the early stage, only failure records from 1990 to 2019 are analyzed to ensure the accuracy.

The historical daily minimum temperature and daily precipitation are selected from the NOAA dataset. The daily minimum temperature is used because water pipe is more sensitive to the low temperatures based on the field observations by experienced engineers at the Cleveland Water Division. The unit for the temperature is Celsius (°C), and the unit for the precipitation is millimeters (mm). Previous studies have found the time-lag effects of climate on the pipe failure, since pipes are buried underground^17^. In this study, we considered the climate data 30 days before the failure occurred as recommended by^15,17^. From our analyses, this time span also shows a more coherent trend with water pipe failures, whereas use of the other periods results in more scattered results. The moving average values were computed for both the daily minimum temperature and daily precipitation over 30 day period before water pipe failed (Eqs. 1–2).

1 $${\overline{T} }_{d}=\frac{1}{30}\sum_{i=0}^{29}{T}_{d-i}$$

2 $${\overline{P} }_{d}=\frac{1}{30}\sum_{i=0}^{29}{P}_{d-i}$$

where ${\overline{T} }_{d}$ indicates the average temperature value of day *d*,${T}_{d-i}$ is the daily minimum temperature on the ith day before day *d*, ${\overline{P} }_{d}$ indicates the average precipitation value for the day *d*. ${P}_{d-i}$ is the daily precipitation on the ith day before day *d*.

It is known that the climate models and projections for the future have range of uncertainties. Therefore, multiple climate models need to be considered to reduce and account for the uncertainty^25^. We considered three models for the future climate data, namely the community earth system model version 2 (CESM2), the Canadian earth system model version 5 (CanESM5), and the sixth version of the Model for Interdisciplinary Research on Climate (MIROC6)^26–28^. These models are selected based on the recommendation of a recent publication^6^. For each model, three Shared Socioeconomic Pathways (SSPs) are considered, i.e., SSP1-2.6, SSP3-7.0, and SSP5-8.5. The different SSPs represent the different future socio-economic assumptions^29^. For example, the SSP1-2.6 represents the scenario when global CO_2_ emission is cut significantly, and the anticipated global warming is less than 2 °C at the end of this century. On the other hand, the SSP 5–8.5 represents the high CO_2_ emission to produce a radiative forcing of 8.5 W m^−2^ in 2100. The SSP 3–7.0 is in the middle of these two models, whose anticipated radiative forcing in 2100 is 7 W m^−2^ ^30^. Similar to the data preprocessing in historical climate data, the moving average values (Eqs. 1–2) are computed for the future climate.

### Single-factor failure rate model

**Figure 3:**
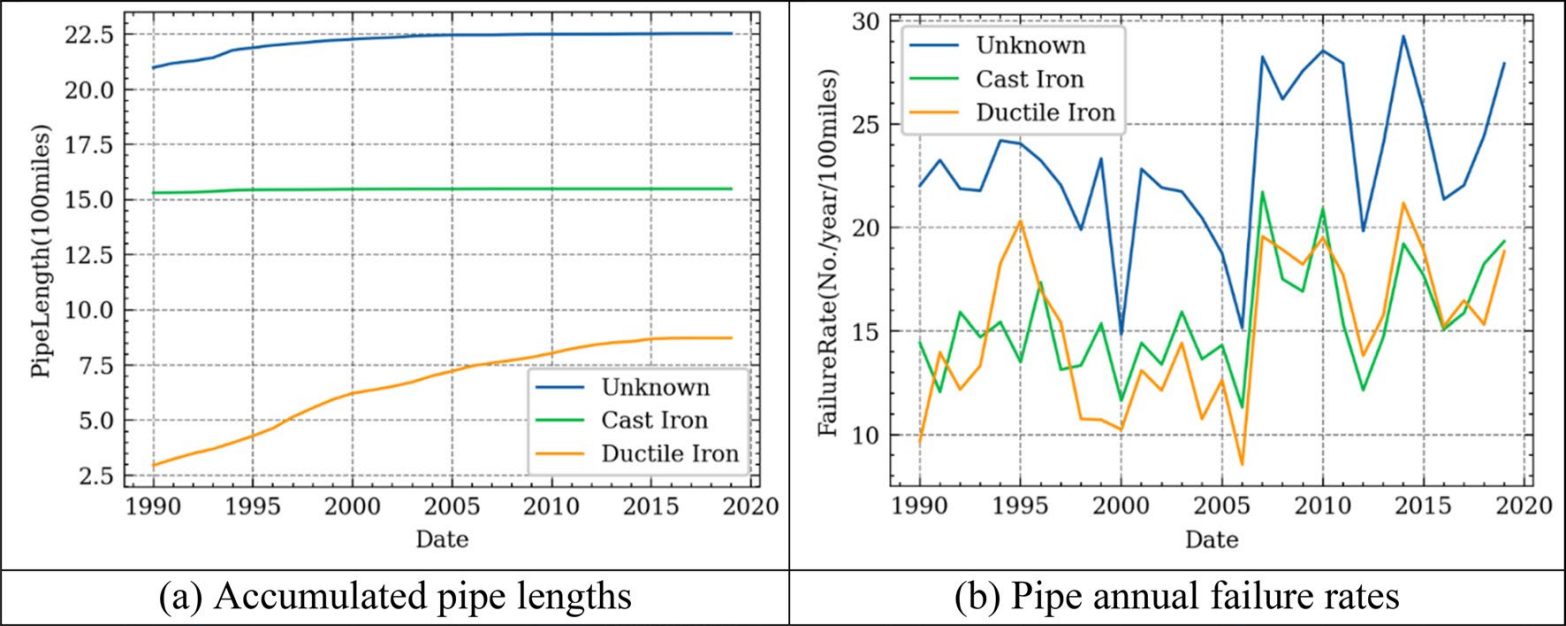
Historical pipe lengths and failure rate of different materials.

The standard unit for the failure rate or failure frequency in the water system is the number of failures (or failure numbers) per unit time per unit length^31^. In this study, we defined the failure rate as failure numbers/day/100 miles. We studied three different failure rate models respectively, i.e., the pipe’s failure rate model for ages, temperature, and precipitation. It is important to note that the pipe lengths increased annually for the Cleveland water system in the past 30 years, as shown in Fig. 3(a). Especially for the ductile iron pipes, whose lengths have significantly increased from around 250 miles to 800 miles. The lengths of pipes at each age also dynamically changed each year when considering the pipes’ ages. Therefore, it is important to compute the pipe failure rates considering the temporal influence. Based on the definition of failure rate, the historical failure rates of pipes made of different materials are shown in Fig. 3(b). It shows that while cast iron pipes typically have higher failure rates than the ductile iron pipes before 2005, in the years since then their failure rates are generally comparable. This is possible because most ductile iron pipes are in the relatively young stage (below 25 years old) before 2005. The earliest installation date of the ductile iron pipes is 1960, most were installed between 1980 and 2000. The pipes with ‘unknown’ material have the higher failure rates compared to the pipes made of cast iron or ductile iron materials. This is possibly because the ages of pipes with unknown materials are older.

**Figure 4:**
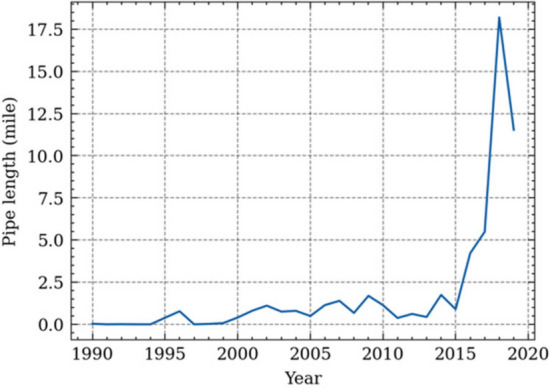
Pipe lengths of 30-years ductile iron pipes at each year.

Equation 3 shows the failure rate equation of different pipe ages. The failure rate is computed for each year by dividing the failure numbers by the total pipe length at that age. It should be noted that there may not have sufficient pipe samples at a specific age for certain years. For example, there only existed a few ductile iron pipes older than 30 years before 2000 as shown in Fig. 4. It is because ductile iron pipes were only used since 1970. The years before 2000 will not give representative samples to analyze. Therefore, only the years with sufficient number of pipe samples are considered valid in the analyses. In this study, the valid years for a specific pipe material and age group is defined as the years when the total pipe lengths of pipe made of that material and within the specified age group are larger than 1 mile.

3 $$F{R}_{a}=\frac{1}{N}\sum \left(\frac{F{N}_{a}^{n}}{{L}_{a}^{n}}\right)$$

$$n\in \left(1990, \dots ,2019\right)\,if\,{L}_{a}^{n}>1\,mile$$

where $F{R}_{a}$ is the failure rate at age *a*, $F{N}_{a}^{n}$ is the failure number of pipes of age *a* at year *n*, ${L}_{a}^{n}$ is the total length of pipes of age *a* at year *n*. *N* is the number of valid years.

The failure rate models of temperature or precipitation are studied under different pipe age brackets. Four age brackets are used so that enough pipe samples fall into each age group, i.e., 0 to 25 years, 25 to 50 years, 50 to 75 years, and 75 to 100 years. With three materials and four age brackets, a total of 12 pipe groups are partitioned. The climate factors, i.e., the temperature or precipitation, are divided into 10 brackets over their typical ranges. In this study, the temperature ranges from −10 to 20 °C, and the precipitation ranges from 0 mm to 5.5 mm. The pipe age and climate brackets are determined in a trial-and-error process to minimize abnormal failure rates. A smaller range of brackets would decrease the number of samples belonging to each bracket and therefore increase the probability of outliers. A wider range of brackets would decrease the generality of the proposed model.

Equations 4 and 5 are used to calculate the pipe failure rate of a specific age-material group at the specific climate bracket. Similar to calculating the failure rate against pipe ages, the failure rate must be computed annually since the total length of the pipe system changes over years. The failure rate of a specific group of pipe each year equals dividing the number of failures by the number of days in that climate bracket and then by the total length of the pipes (Eq. 4). It is noted that the cohort groups analyzed include different site-specific characteristics. Therefore, the influences of these site-specific factors, such as the accidents, soil types, etc. are averaged in certain sense. The study primarily focuses on the influence of climate factors. Fan et al. (2022) describes the analyses of the influence of engineering, geology, climate and socio-economic factors on pipe failures.

4 $$F{R}_{\left(t, a\right)}^{n}=\frac{F{N}_{\left(t,a\right)}^{n}}{{D}_{t}^{n}*{L}_{a}^{n}}$$

where $F{R}_{\left(t, a\right)}^{n}$ is the pipe failure rate at temperature bracket *t* and age bracket *a* at year *n*, $FN$ is the number of pipe failures, *D* is the number of days, *L* is the total length of pipes. *t* is the temperature bracket whose unit is °C. The failure rate of precipitation can be calculated similarly by replacing the *t* with precipitation *p*.

Also, considering the influence of insufficient pipe samples, only the years whose total pipe length is larger than 25 miles are considered the valid year. Be aware that this threshold is larger than that used in the failure rate mode for the effects of age. This is because the failure rate model of climate considers a 25 years-period. This threshold is selected to guarantee that the recorded failures are observed in at least five climate brackets. Lastly, the final failure rate at a specific climate bracket and age bracket is the weighted mean value of the failure rate at each year (Eq. 5).

5 $$F{R}_{\left(t,a\right)}=\sum_{n}\left(F{R}_{\left(t,a\right)}^{n}*{w}_{t}^{n}\right)$$

6 $${w}_{t}^{n}=\frac{{L}_{t}^{n}}{{L}^{t}}$$

$$if\,{L}_{a}^{n}>25\,miles$$

where *N* is the number of validated years. A validated year means the total length of the pipe group at this year is larger than 25 miles.

### Pipe failure fragility over climate

**Figure 5:**
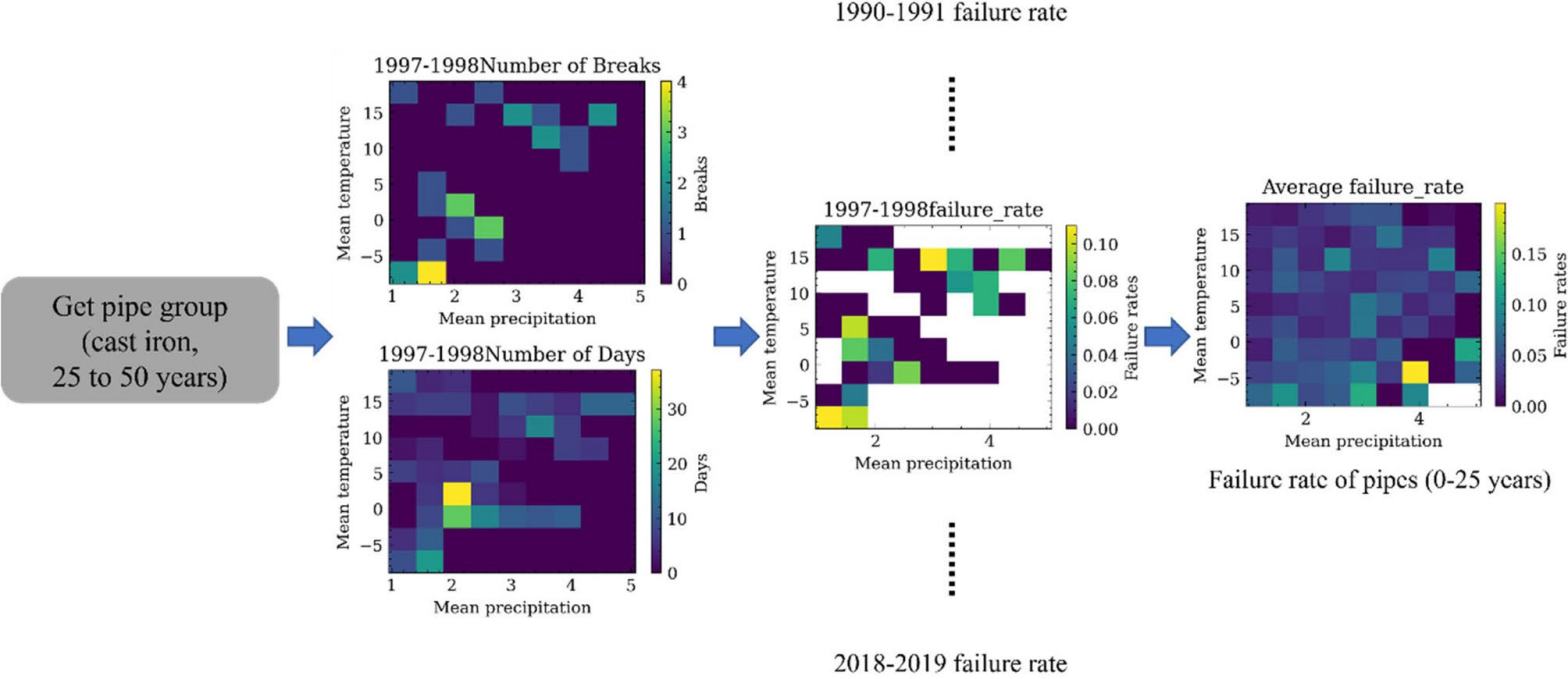
Illustration of the failure rate map (or fragility over climate) for cast iron pipes with age between 25–50 years.

It is known that both climate factors (i.e., temperature and precipitation) impact the water system’s failure frequency. However, previous failure rate models, especially statistical ones, were only developed to consider one climate factor^11^. Single-factor failure rate models may not be enough for prediction of future climate effects since the climate change will alter both the temperature and precipitation patterns. In this study, we computed the failure rates of the water pipes, or climate fragility of water pipes, considering both temperature and precipitation. The concept of failure rate map was used. Figure 5 illustrates the computing process of computing the 0–25 years cast iron pipe’s climate fragility, which utilized 1997–1998 to illustrate. For each year, the pipes belonging to a specific pipe group were first selected. Then, the climate days and number of failures were partitioned into each temperature and precipitation brackets. The annual failure rate is computed by dividing the number of breaks by the number of days and the total length of the pipe group (Eq. 7).

7 $$F{R}_{\left(t,p,a,n\right)}=F{N}_{\left(t, p, a,n\right)}/{D}_{\left(t,p,n\right)}/{L}_{n}$$

where the $F{R}_{t,p,a,n}$ is the failure rate of pipes at age *a* when temperature at* t* and precipitation at *p* for year *n*. The FN, D, and L are the same as (Eqs. 4–5).

The final climate fragility map (which indicate the failure rates versus climate) are computed by the mean value of the climate fragility map for valid years between 1990 to 2019 (Eq. 8).

8 $$FR\left(t,p, a\right)=\frac{1}{N}\sum FR\left(t,p,a, n\right)$$

where *N* is the total number of valid years.

Many specific combinations of temperature and precipitation have not been recorded in the historical data, but a regression model allows to recognize the probable outcomes for those inputs. Thus, a regression model is necessary for computing the impacts of climate change in the long-term future^11^. The Kriging algorithm is a spatial interpolation technique that has been widely used for geospatial and hydrogeologic discipline analysis^32^. It estimates the unknown value by using the weighted average of nearby samples. Therefore, it simultaneously considered the spatial locations in the regression process. The kriging algorithm is demonstrated to provide the best linear unbiased estimator in the perspective of statistical^33^ for a 2D spatial interpretation.

Different Kriging models have been developed in previous studies^34^. The Ordinary Kriging algorithm is firstly explained in the following. As shown in Eq. 9, it estimates a location's value by using the weighted sum. The weight of each point, ${\lambda }_{i}$, is determined by using the semivariograms^35^ and Lagrange multiplier method. The empirical semivariogram used in this study is the sum of squared differences between values separated by a distance *h* (Eq. 10). The key idea of the Kriging algorithm is to minimize the mean squared perdition error of the predicted values. The detailed calculation process of the Ordinary kriging algorithm can be referred to Van Beers and Kleijnen^36^.

9 $$\widehat{Z}\left({s}_{0}\right)=\sum_{i=1}^{N}{\lambda }_{i}Z\left({s}_{i}\right)$$

where $\widehat{Z}\left({s}_{0}\right)$ is the predicted value at ${s}_{0}$, ${\lambda }_{i}$ is the weight for the measured value at climate bracket *i*. $Z\left({s}_{i}\right)$ is the measured value at *i* location. N is the number of measured values.

10 $$\widehat{\gamma }\left(h\right)=\frac{1}{2N\left(h\right)}\sum_{\alpha =1}^{N\left(h\right)}{\left({z}_{{u}_{\alpha }}-{z}_{{u}_{\alpha }+h}\right)}^{2}$$

where N(h) denotes the number of pairs of sites separated by a distance h. ${z}_{{u}_{\alpha }}$ and ${z}_{{u}_{\alpha }+h}$ are two points that are separated by the distance *h*.

However, the Ordinary Kriging requires a stationary assumption which means the mean and variance values is constant across the study space^37^. Considering a trend may exist among the failure rate and climate factors, the Universal Kriging method is believed to get a more mathematically accurate result since it could consider the trend inside the dataset^38^. The Universal Kriging is very similar to the Ordinary Kriging model, except a drift model is added. In this study, the regional-linear drift model proposed by^39^ is used. The variance of the Universal Kriging estimator at the location (${s}_{0}$) is shown in Eq. (11) ^40^. The final prediction function $\widehat{Z}\left({s}_{0}\right)$ and semivariogram function $\widehat{\gamma }\left(h\right)$ are the same to the Simple Kriging. The PyKrige toolkit is used in this study for the model computation process^41^.

11 $${\sigma }^{2}=\sum_{i=1}^{N}{\lambda }_{i}\gamma \left({s}_{0}-{s}_{i}\right)+\omega +{\beta }_{1}{x}_{0}+{\beta }_{2}{y}_{0}$$

where ${\sigma }^{2}$ is the variance of the Kriging estimator, $\omega , {\beta }_{1},{\beta }_{2}$ are Lagrange multipliers.

### Simulation and comparison of climate change impacts on water supply system

**Figure 6:**
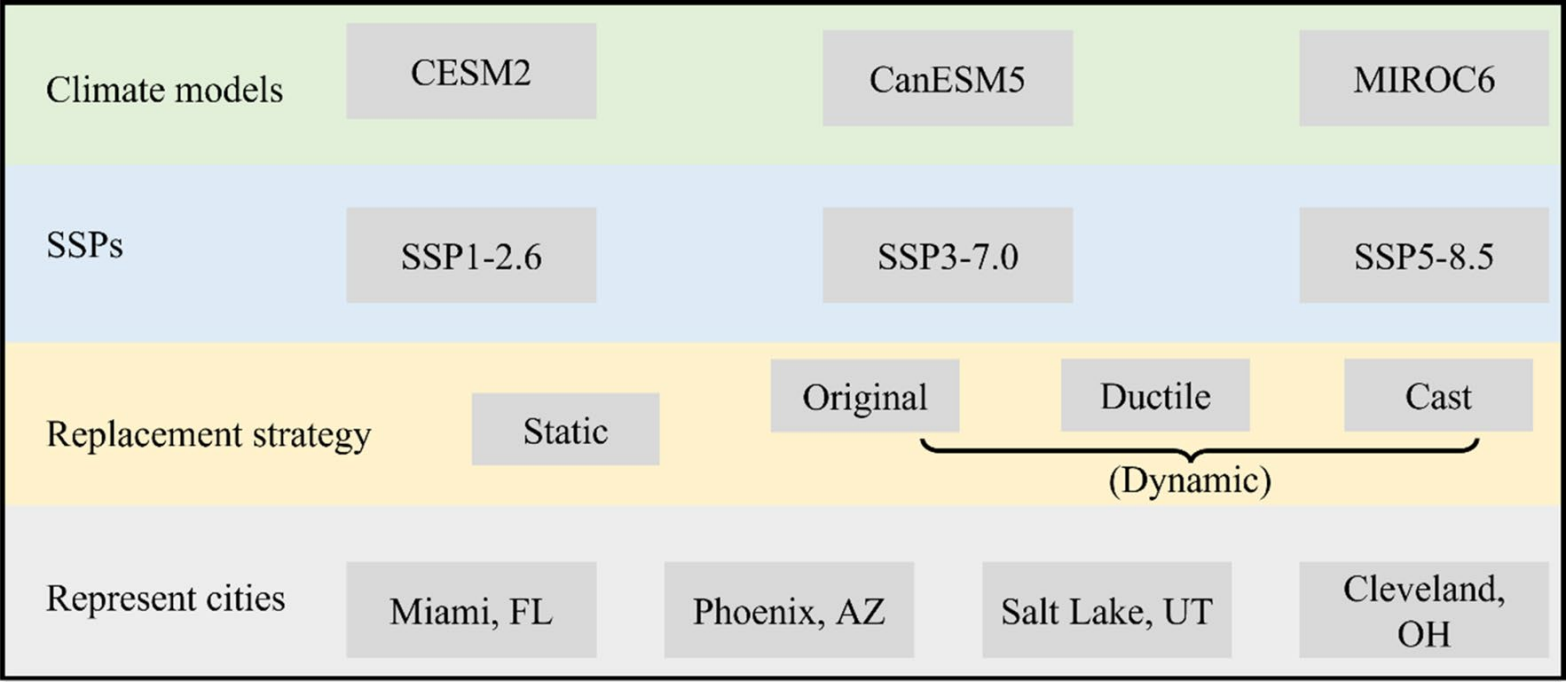
Considered scenarios for comparison purpose.

The impacts of climate change on the water system are simulated under multiple scenarios, which include the climate change, replacement strategy, geographic location, as shown in Fig. 6. Three climate models were used to simulate the future climates under three different Shared Socioeconomic Pathways (SSPs) to consider the effects of uncertainties of prediction models and social-economic development paths. Two types of pipe replacement strategies for the water supply system, i.e., the static replacement and dynamic replacement, were analyzed over the period of 2020–2100 to understand the long-term influence of management policies. The static strategy assumes the pipe ages and materials of the water system are the same as 2020 in the following 80 years. This assumption, while not representing the field aging process, is made to study the impacts by climate change only. The dynamic strategy assumpes the pipe information is continuously updated by replacing the aged pipes with different materials. Pipes are assumed to be replaced when they reach 100 years old. For the materials of replacement pipes, the ‘Original strategy’ assumes the pipes at the end of service life are replaced with pipe with the same original materials. The ‘Ductile strategy’ assumes the aged pipes are replaced with Ductile Iron pipes, and the ‘Cast strategy’ assumes the aged pipes are replaced with the Cast Iron pipes.

It is noted water pipe replacement strategies assumed in this study is much simplified than the real world scenarios. This is because that the analyses here primarily aim to examine the potential impacts and consequences of climate change and pipe aging. The simplifications allow to focus on these two primarily factors.

Four representative cities are selected from different climate zones^42^. The climate conditions for the selected cities are classified as hot and humid, hot and dry, cold and dry, and cold and humid, respectively.

The failure rate and failure numbers for the water system located in the selected cities were computed for the next 80 years after 2020. The computation considers climate models, SSPs, replacement strategies, and locations. For each year, the failure rate of a specific material is computed by Eq. (12). It is a weighted sum of the failure rates of pipes in different age groups. The weights are determined by the proportion of the specific age group to the total material length at year *n*. The failure rate of pipes at a specific age and material group is the sum of their daily failure rates based on the daily forecast temperature and daily precipitation. Hence, the failure rate in the unit of failure numbers per year per 100 miles is determined.

12 $$FR\left(n,m\right)=\sum_{\mathrm{a}}\left(\frac{{L}_{(a,n,m)}}{{L}_{n,m}}\right)\sum_{d=1}FR\left(a, m, {t}_{d}, {p}_{d}\right)$$

where $FR\left(n,m\right)$ is the failure rate of pipe made of material type *m* at year *n*, the *m* includes the cast iron, ductile iron, and unknown materials, and the *n* ranges from 2020 to 2100. ${L}_{(a, n,m)}$ is the length of pipe made of material *m* at age bracket *a* of year *n*. The range of *a* is the four age brackets, i.e., 0–25, 25–50, 50–75, and 75–100. The ${L}_{(n,m)}$ is the total length of material *m* at year *n* regardless the ages. $FR\left(a, m, {t}_{d}, {p}_{d}\right)$ is the failure rate of pipe made of material *m* and age group *a* at day *d* whose temperature and precipitation are ${t}_{d} and {p}_{d}$. The day *d* ranges from 1 to 365 (or 366) of the corresponding year.

The annual failure number of pipe made of a certain material is its failure rate multiplied by the total length of pipe made of this material, as shown in Eq. (13). The water system’s total failure numbers can be computed by the sum of failure numbers of pipe made of each material.

13 $$FN\left(n,m\right)=FR\left(n,m\right)*{L}_{n,m}$$

where $FR\left(n,m\right)$ is the failure rate of material *m* at year *n* as computed in Eq. (12). The ${L}_{n,m}$ is the total length of material *m* at year *n*.

## Results and discussion of failure rate models

This section represents the failure rate models of single and multi-climate factors. It only illustrates the pipe age and climate impacts on the pipe failures without considering the climate change in the next 80 years. "Evolution of pipe failure rate with age for pipes made of different materials" section shows the failure rate model of pipe ages. Then followed by the failure rate model of temperature and precipitation, respectively ("Pipe failure rate versus temperature and precipitation" section). "Failure rate fragility map over both temperature and precipitation simultaneously" section shows the multi-climate model by using the failure rate maps.

### Evolution of pipe failure rate with age for pipes made of different materials

**Figure 7:**
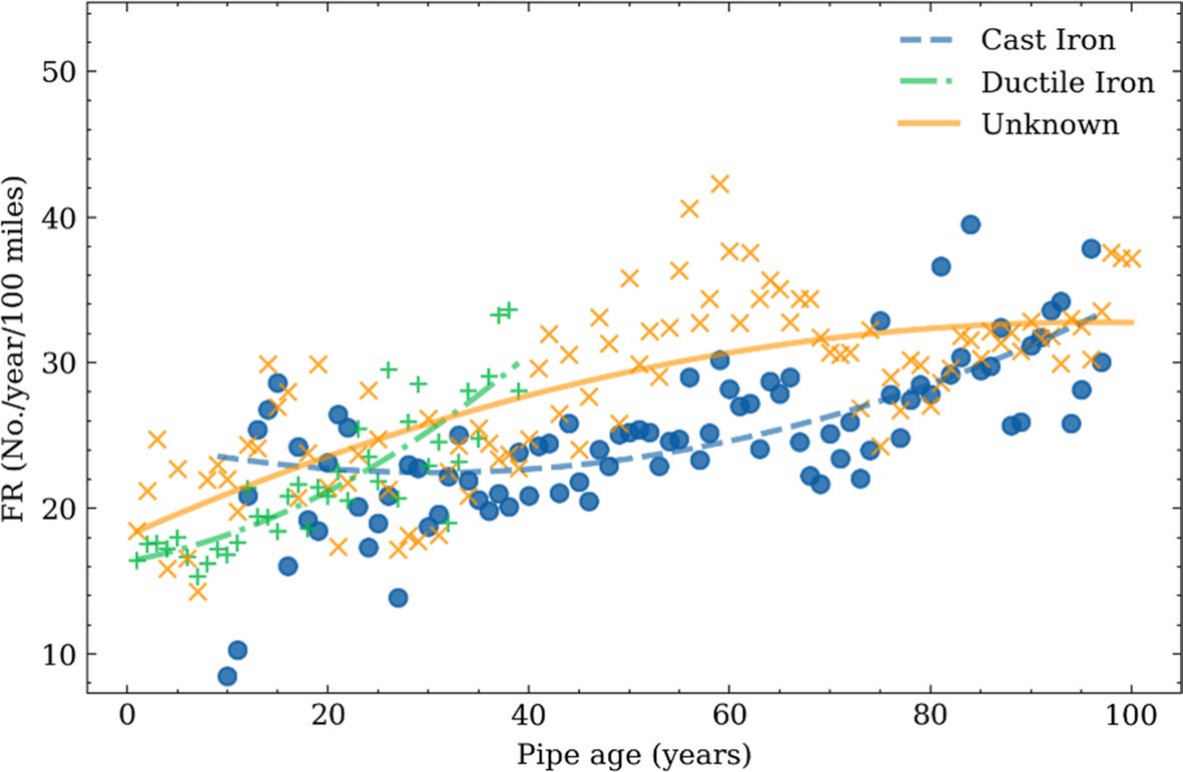
Failure rates at different pipe ages.

Figure 7 shows the evolution of failure rates of pipes made of different materials with age computed by use the method described in "Single-factor failure rate model" section. Data points of pipe failure rates stopped at around 50 years for the ductile iron pipe because ductile iron only began to be used in water systems in the 1960s. The other types of pipes have been used for more than 100 years and thus have failure rate data over 100-year time span.

A quadratic curve was used to fit the failure rate trends (i.e., Eq. 14). Table 1 shows the parameters and performance of curve-fitting results. The root mean square error (RMSE) is used to evaluate the goodness of fitting (Eq. 15).

14 $$FR=\alpha *{a}^{2}+\beta *a+\sigma$$

where *a* is the pipe age, $\alpha$, $\beta$, and $\sigma$ are the model parameters.

15 $$RMSE=\sqrt{\frac{{\sum }_{i=1}^{N}{\left({x}_{i}-{\widehat{x}}_{i}\right)}^{2}}{N}}$$

where *N* is the total number of samples, ${x}_{i}$ is the true value of sample *i*, and the ${\widehat{x}}_{i}$ is the estimated value of sample *i*.

**Table 1:** Failure rate models of the pipe ages.

Material	$\alpha$	$\beta$	$\sigma$	RMSE
Cast iron pipe	0.002	−0.149	24.720	5.748
Ductile iron pipe	0.006	−0.125	16.364	2.433
Unknown pipe	−0.002	0.304	18.111	4.019

A ‘bathtub’ trend is observed for the cast iron pipes, indicating a higher failure rate during the early stage as well as late stages of pipe service life. Similar trends have been reported in previous studies. The higher failure rate at the early stage is possibly caused by non-uniform ground settlement after pipe installation combined with that cast iron pipe is brittle and prone to crack. Once the ground settlement accomplished, the failure rates decrease and maintain constant^43^. A higher failure rate appears at the late stage of cast iron pipes, mainly caused by the pipe aging and deterioration under the effects of service loads, corrosion etc.

Pipes made of ductile iron show a relative low and stable failure rate compared with cast iron pipes. And then they gradually increase failure rates with age. There are significant scattering in the failure rate data for pipes over 30 years old, while overall it shows an increasing trend. This observation is counterintuitive to the general belief that pipe failure rate becomes stable after long service. This is possible because that ductile iron pipes are mostly less than 50 years old, indicating fewer samples compared to the other pipes.

From the results shown in Fig. 7, cast iron pipes have a higher failure rate than the ductile iron pipes during the early stage of service; they feature lower failure rate than ductile iron pipes after around 30 years old. The former observation is consistent with many previous studies, which indicated that the ductile iron pipes are more flexible and have a better ability to resist the failure due to soil settlement^44,45^. However, the latter observation indicates that the ductile iron pipes have a higher failure rate than the cast iron pipes after 30 years old, which has not been reported in the current studies. The reason behind it deserves future research. It should be noted that the cast iron pipes are used much earlier than the ductile iron pipes in the studied dataset. Most ductile iron pipes were installed around 1970 and aged between 35 to 50. The documented failure records are after 2010; whereas the cast iron pipes with similar ages were installed after 1940 with documented records are between 1990 and 2019. There might be differences due to installation methods, environmental conditions, and water service conditions, etc.

The overall failure rates For pipes with unknown materials increase with age and becomes relatively stable.

### Pipe failure rate versus temperature and precipitation

**Figure 8:**
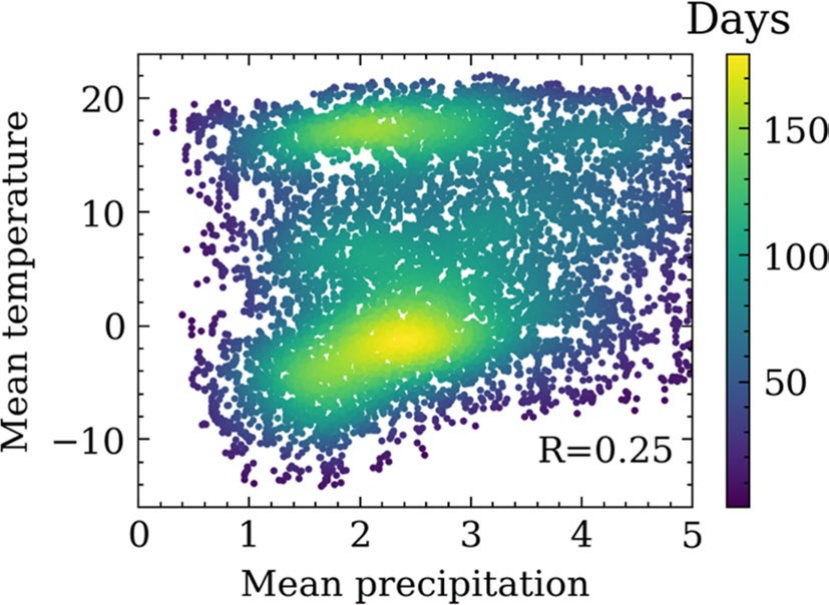
Statistic distribution of the mean daily temperature and precipitation in cleveland, Ohio, USA between 1990 to 2019.

Figure 8 shows the statistics of daily average temperature and total precipitation in Cleveland during the past 30 years. The color code indicates the density of the days that belong to the climate combinations of a certain daily average temperature and precipitation. That is, the more days fall into a specific temperature-precipitation combination, the deeper the color is. Figure 8 shows that in Cleveland weather, temperatures typically fell between 0 and 20 °C with precipitation range from 1 to 3 mm per day. However, there are some data points with temperatures below 10 °C. No strong correlation has been observed between the average daily temperature and total daily precipitation from the historical climate data, with the correlation coefficient between the daily temperature and precipitation only about 0.25, as denoted in the right bottom of Fig. 8. The low correlation between the temperature and precipitation is possibly due to the unique geographical location of Cleveland, as the city is located along the southern shore of Lake Erie. Hence the dataset is ideal for analyzing the impact of temperature and precipitation independently.

**Figure 9:**
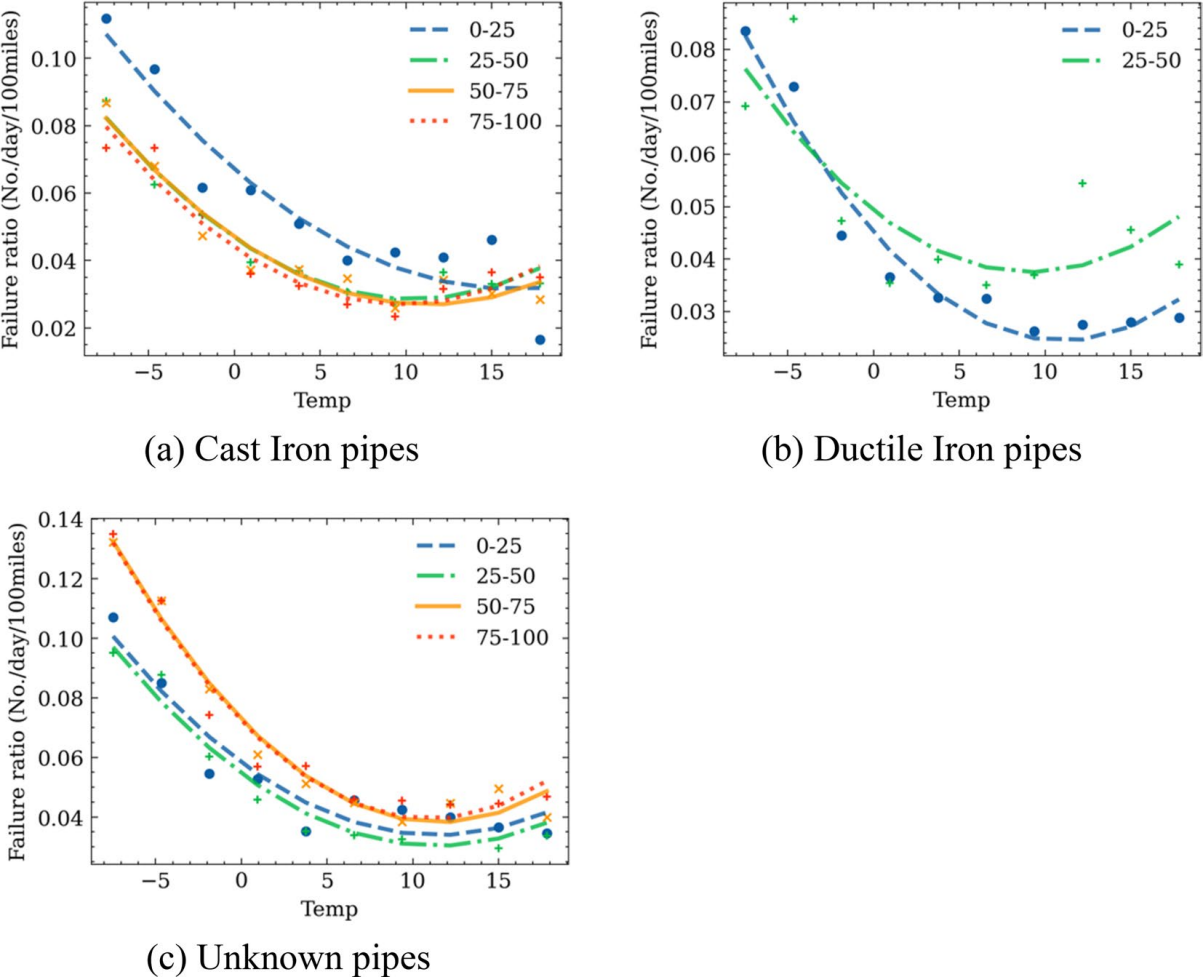
Failure rate versus temperatures for pipes made of different materials and in different age groups.

**Figure 10:**
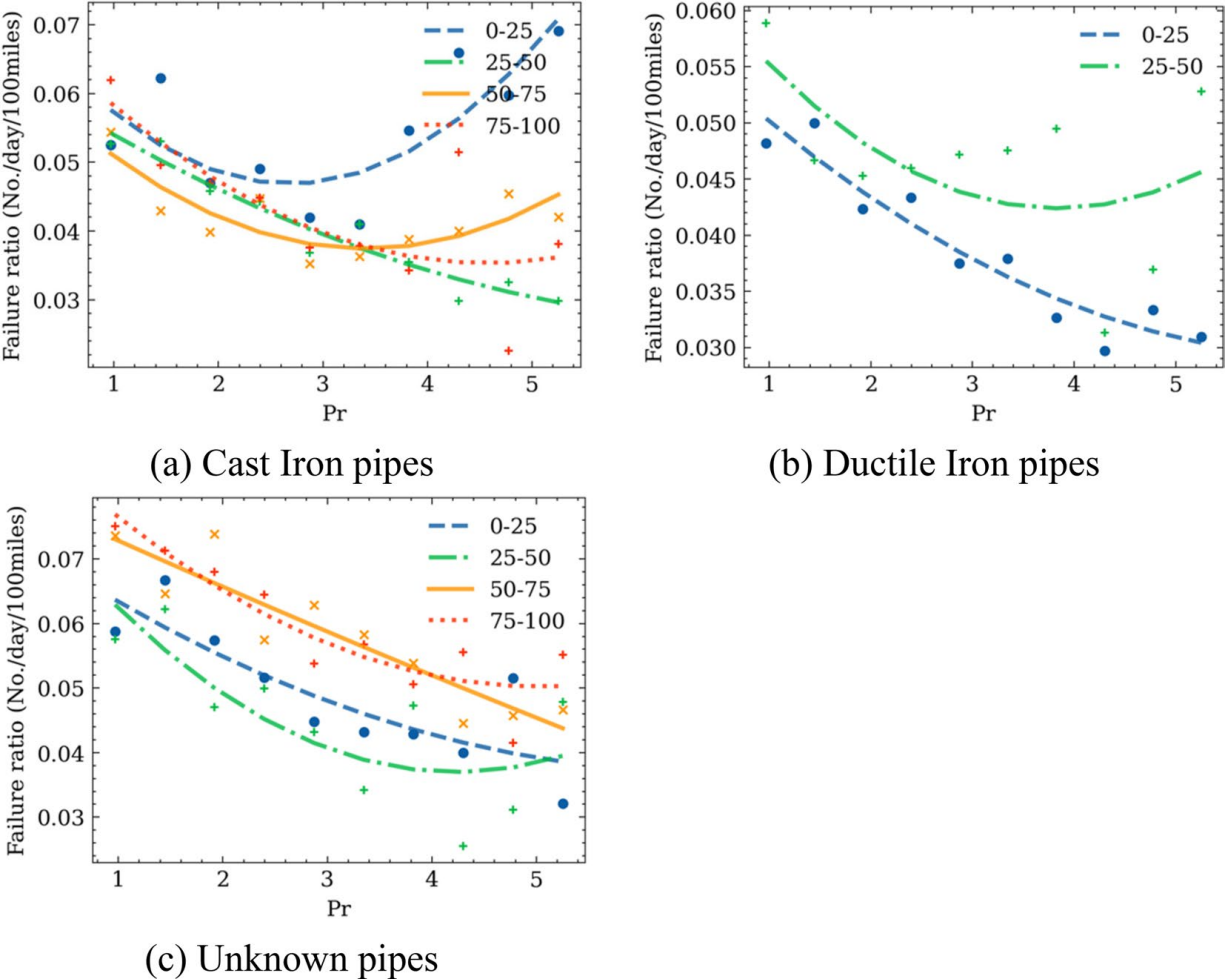
Failure rate versus average daily precipitation for pipes made of different materials and in different age groups.

The fragility curves over temperature and precipitation, i.e., the failure rates versus temperature or precipitation, are developed for pipes made of different materials at different age groups. Pipes are categorized into different age groups every 25 years. For each temperature or precipitation bracket, the middle value and the corresponding failure rate are obtained and are plotted in Figs. 9 and 10, respectively. To account for the effects of climate history, the mean value of temperature and precipitation over 30 day period before the date when a pipe failure occurred were used. This number of days is selected based on the recommendation in previous studies as well as test run process during this study. Unlike previous studies that used a linear regression model to fit the failure rate points^1,3^, a parabola shape is observed in the fragility curves (Figs. 8 and 9), which is approximated with quadratic polynomial equations. Table 2 shows the corresponding models, and the root mean square error (RMSE) between the predicted value and the ground truths (Eq. 15).

**Table 2:** Failure rate models of temperature brackets.

Material	Age	$\alpha$	$\beta$	$\sigma$	RMSE
Cast Iron	0–25	0.00013	−0.0044	0.067	0.009
	25–50	0.00017	−0.0035	0.047	0.003
	50–75	0.00016	−0.0035	0.047	0.005
	75–100	0.00018	−0.0035	0.044	0.005
Ductile Iron	0–25	0.00017	−0.0038	0.045	0.004
	25–50	0.00014	−0.0026	0.049	0.011
Unknown	0–25	0.00019	−0.0043	0.058	0.007
	25–50	0.00019	−0.0043	0.055	0.006
	50–75	0.00026	−0.0060	0.073	0.005
	75–100	0.00027	−0.0059	0.072	0.006

Figure 9 indicates that the failure rates increase with decreasing temperature regardless of the pipe materials and age groups. Lowest temperature corresponds to the highest failure rates. This is consistent with empirical observations that more pipe failures occurred during cold winter season.

Previous studies indicated that the high failure rates are caused by soil freezing, expansion-related ground movement, and additional compression on the pipes^7,20^. From Fig. 8a, the temperature-fragility curves of young cast iron pipes (0–25 years) have higher failure rates compared to old cast iron pipes, especially under cold winter with low temperatures. There are no significant differences in the temperature-fragility curves of cast iron pipes in different age groups (i.e., between 25–50, 50–75. 75–100 years). This may be caused by the combined effects of ground settlement after pipe installation and freezing soil expansion, and the young cast iron pipes are more vulnerable to displacement.

The results in Fig. 8 also show that increasing failure rates occurred on days with average temperature above 10 °C so so, especially the trend is more obvious for older pipes. The possible reason is that the high temperature on hot weather might cause ground soil shrinkage and accelerate pipe corrosion. Older pipes are more vulnerable to such effects as their remaining yield strength is close to the external load^46^. Besides, hot summers typically cause higher water consumption that may also lead to the higher failure rates, as the internal water pressure of the water system is higher than for the other seasons. Among all the pipe material and age groups, the group of pipe with unknown material pipes have the highest failure rates at high temperatures, which is followed by the older ductile iron pipes. Both young ductile iron pipes and cast-iron pipes show relatively low failure rates at high temperatures.

Finally, regardless of the materials for the water pipes, the lowest failure rates occur on days with average air temperature between 8 to 12 °C. Previous studies have also demonstrated that the Spring and Autumn seasons have the lowest pipe failure rates because the soil moisture is consistent, which decreases the soil movement^7^. The temperature in the middle of spring and autumn are also mild.

Figure 10 shows the impact of precipitations on the pipe failure rates. An overall decreasing trend is observed. The higher failure rates on days with low precipitation are probably caused by the soil shrinkage and swell effects due to the moisture change^47^. However, an increasing trend with precipitation is observed for new cast iron pipes. A probable reason is that the ground deformation has not become stabilized for newly installed pipes and , which makes them more susceptible to high precipitation. Moreover, young cast iron pipes are more vulnerable to ground settlements. It is also noted that the number of water pipes between 0–25 years old and the number of days that have high precipitation are relatively small. The combination of these two factors generates a small denominator when calculating the failure rate by us of Eq. (6). This might cause inaccuracy due to sample size. Future analyses will be needed to further validate this observation.

Model by Eq. (14) is used to fit the observed trends in Fig. 9. The results are summarized in Table 3, which shows the corresponding models, and the root mean square error (RMSE) between the predicted value and the ground truths.

**Table 3:** Failure rate models of precipitation brackets.

Material	Age	$\alpha$	$\beta$	$\sigma$	RMSE
Cast Iron	0–25	0.00593	−0.0253	0.072	0.010
	25–50	0.00235	−0.0214	0.082	0.002
	50–75	0.00075	−0.0100	0.063	0.002
	75–100	0.00092	−0.0128	0.071	0.005
Ductile Iron	0–25	0.00138	−0.0143	0.067	0.003
	25–50	−0.00032	−0.0075	0.075	0.007
Unknown	0–25	0.0046	−0.0392	0.122	0.006
	25–50	0.0024	−0.0224	0.086	0.003
	50–75	0.0032	−0.0328	0.126	0.006
	75–100	0.0012	−0.0159	0.094	0.003

### Failure rate fragility map over both temperature and precipitation simultaneously

Climate change will alter the future trends of temperature and precipitation^48^. Predicting the impacts of climate change on water supply system requires a pipe fragility model that can simultaneously consider the effects of both temperature and precipitation.

**Figure 11:**
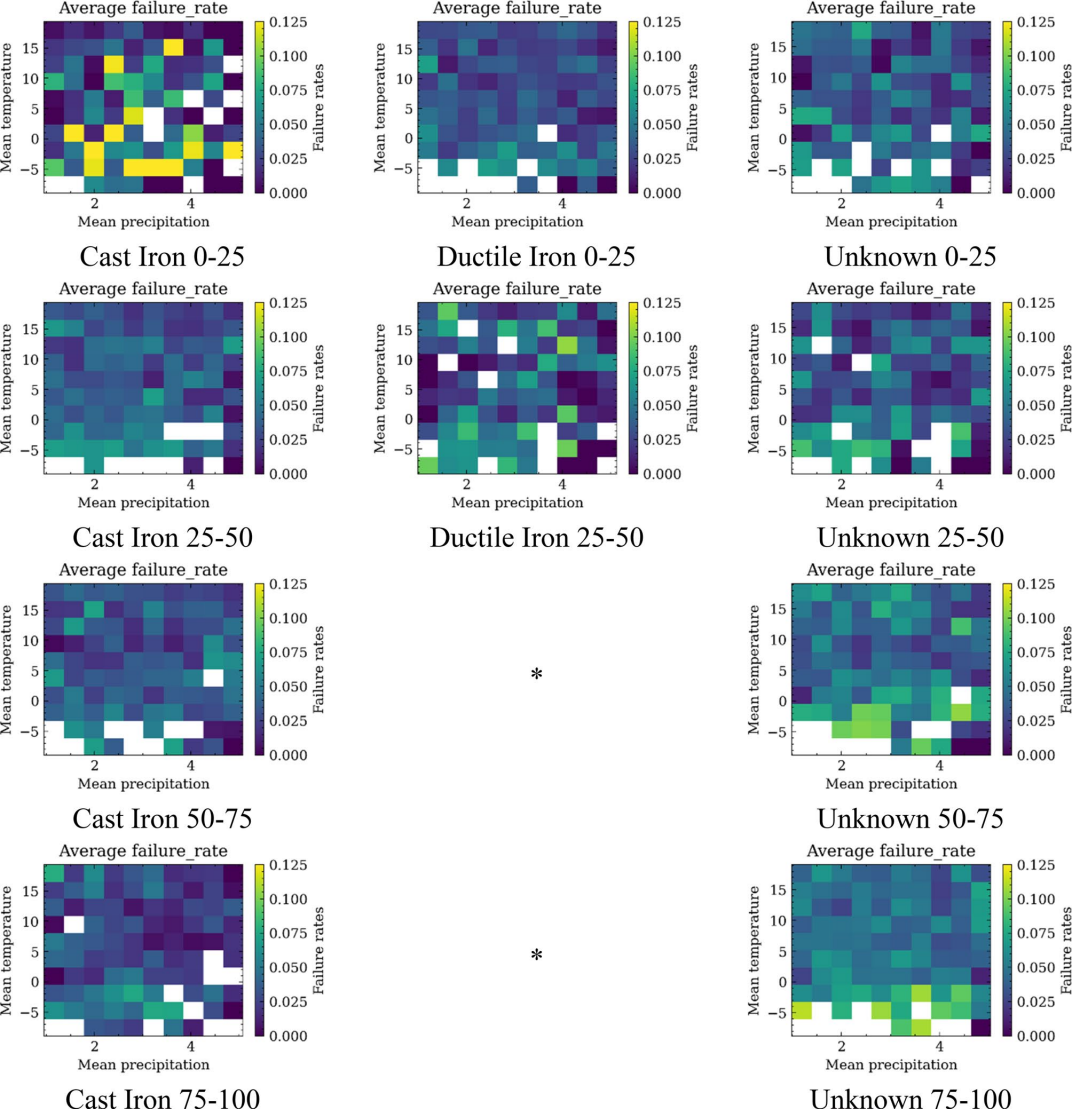
Failure fragility map versus temperature and precipitation for pipes made of different materials and within different age groups.

For this purpose, a 2-D failure rate fragility map is built via the procedures described in "Pipe failure fragility over climate" section. The procedures to build the failure rate fragility map resembles the process of determining the 2-D statistics distribution. The results of 2-D fragility map are shown in Fig. 10 for failure rates of water pipes of different age and pipe groups. The unit for the failure rate is failure numbers/day/100 miles. The unit for the temperature is Celsius and for the precipitation is millimeters. The blank pixels indicate that there are zero days represented by that combination of inputs in the considered validated years (the years that total pipe length is larger than 25 miles). Figure 11 provides a similar but more detailed trend compared to the models in "Pipe failure rate versus temperature and precipitation" section. The results indicate that the failure rate is generally higher during period with low temperature and precipitation. Some high failure rate outliers are observed for specific weather combinations, possibly due to small number of days when pipe failure occurred in the combination of specific temperature and precipitation.

**Figure 12:**
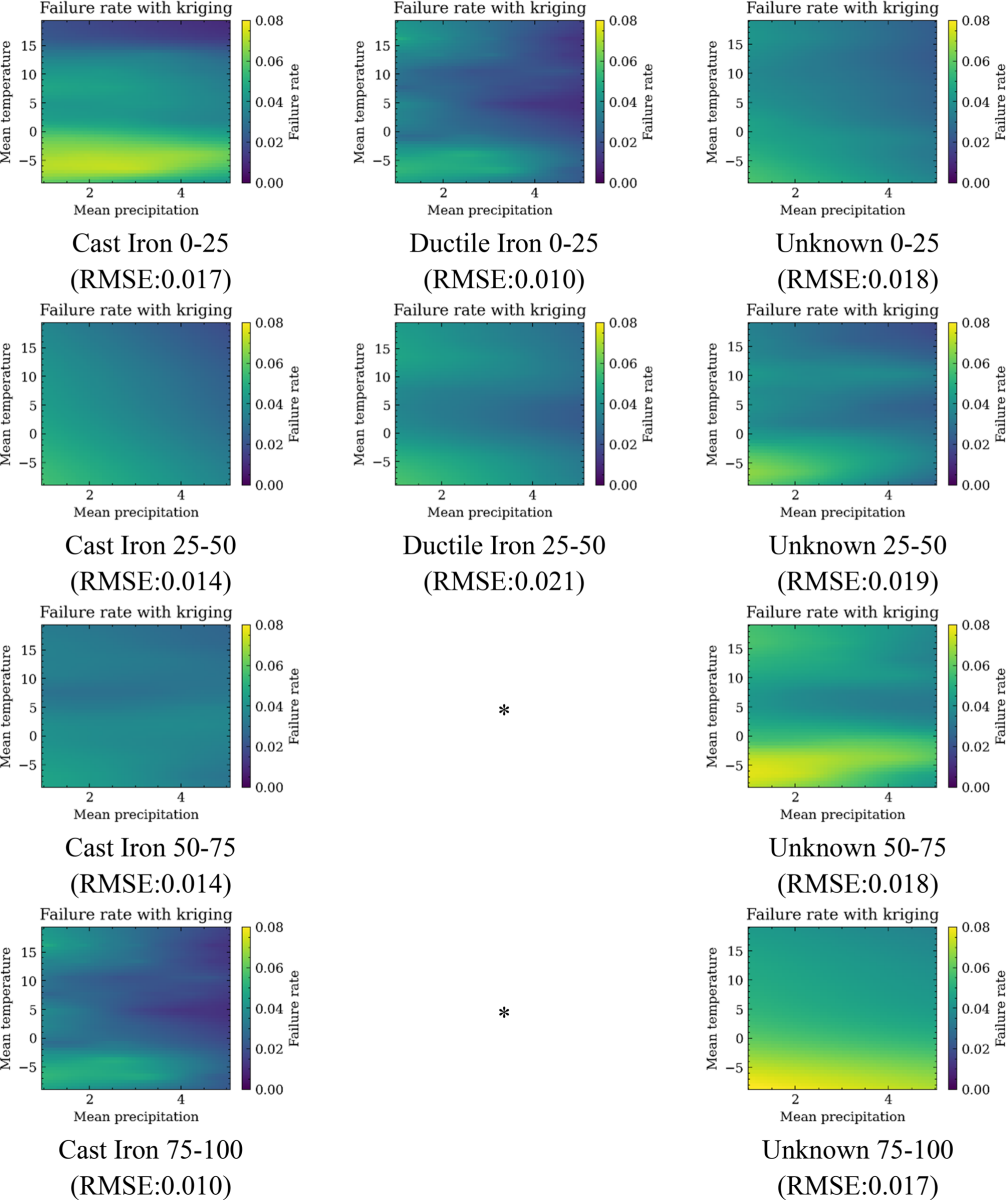
Failure rate map against temperature and precipitation after Universal Kriging (* indicates the failure rate is assumed to be the same to the previous one).

The raw failure rate fragility maps are relatively coarse in the resolution to ensure that there are sufficient samples in each cell to minimize outliers. The Universal Kriging algorithm is used to improve the resolution of fragility map, as discussed in "Pipe failure fragility over climate" section. The results are shown in Fig. 12. The RMSE values between the predicted values and ground truths are also calculated. Since the Kriging algorithm is always faithful to the ground truth, the cross-validation method is used to get the RMSE value^49^. A smooth trend is captured by the Kriging algorithm, which makes the model suitable for studying the impacts of future climate change on the water systems.

## Assessment of the impacts of climate change on water system performance and upgrade

With the water pipe climate fragility models developed in "Failure rate fragility map over both temperature and precipitation simultaneously" section based on data from water system, analyses are conducted on the impacts of climate change on hypothetic water supply system similar as Cleveland water system but located at different climate zones in the United States. The annual failure numbers of the whole water system are computed under different climate change scenarios predicted by different global climate models (GCM) and socio-economic assumptions. Multiple GCMs and socio-economic assumptions are used to account for the uncertainties induced by climate models and maintenance activities. The analyses aim to provide overall assessment on the impacts of climate change on water system conditions and to assist local water agencies in maintenance actions to adapt to the changing climate.

### Considered cities and climate change patterns

**Figure 13:**
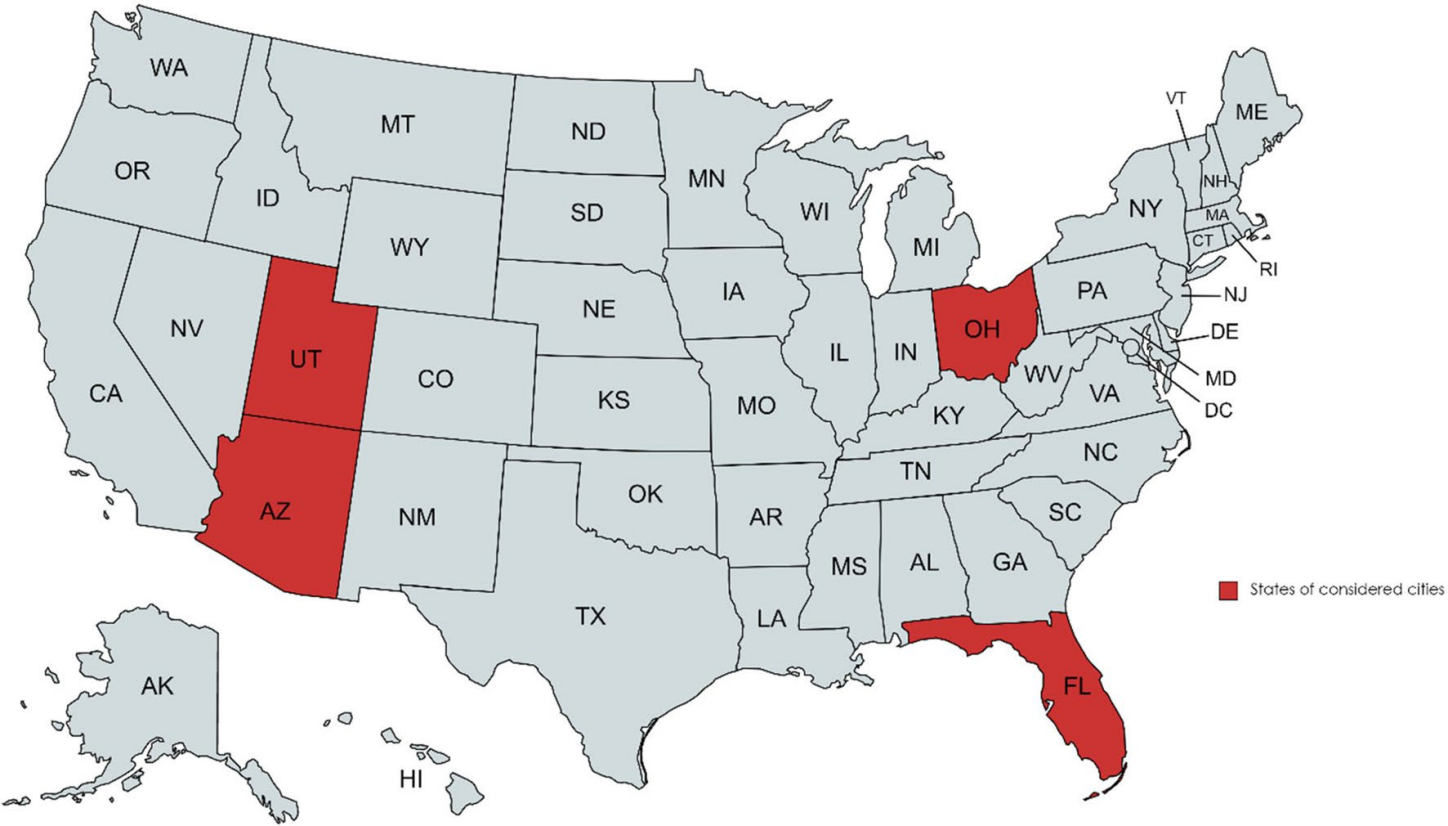
Sates of the selected cities to analyze the effects of climate change on water system (created with MapChart.net (https://www.mapchart.net/)).

Figure 13 shows the locations of the selected cities, i.e., Cleveland, OH, Salt Lake City, UT, Phoenix, AZ, and Miami, FL^42^. Based on the temperature and precipitation, these four cities represent cold-wet, cold-dry, warm-dry, and warm-wet climate conditions. The water system of Cleveland is used for analysis rather than the water system of each city.

**Figure 14:**
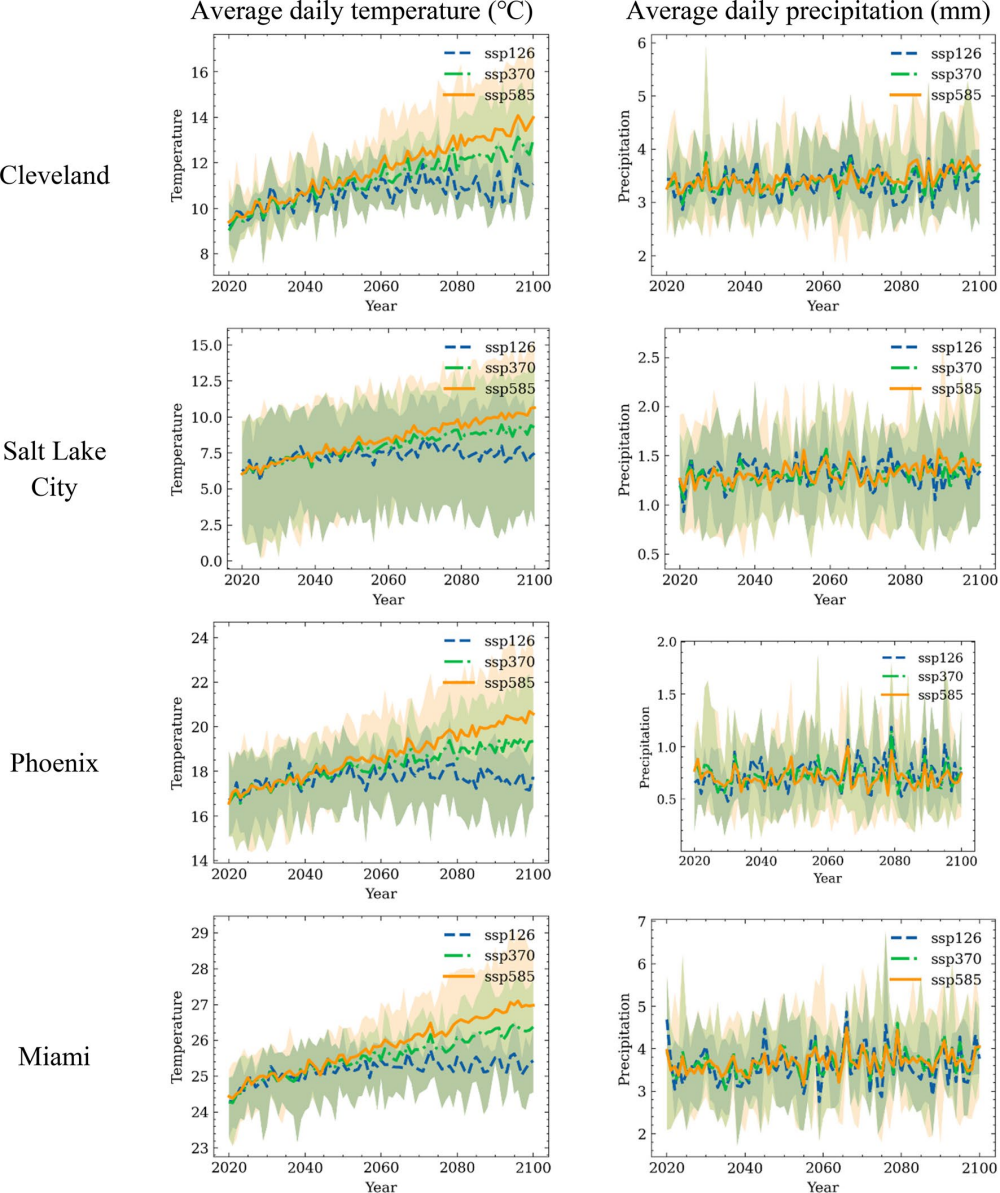
Yearly averaged daily temperature and precipitation at the selected cities.

To consider the effects of climate change, the climate at the four selected cities between 2020 and 2100 are predicted. For each year and SSP, the daily temperature and precipitation are extracted from CESM2, CanESM5, and MIROC6 models. Figure 14 shows the annually-averaged daily temperature and precipitation temperature of the selected cities between 2020 to 2100.

A steadily increasing trend can be observed for the temperature regardless of location and SSPs. The SSP5-8.5 has a higher average temperature than the other SSPs in 2100 and the SSP 1–2.6 has the lowest temperature. Among all the considered locations, Salt Lake City has the largest uncertainty when considering these three different GCMs. The precipitation trend is less significant than the temperature for the considered locations. It is because climate change impacts more on the precipitation intensity change than the total precipitation. The annually averaged value is used here just for better visualization. The precipitation change is expected to be more intense due to climate change^50^.

The number of failures each day is computed using the climate fragility map with temperature and precipitation. From these, the annual failure number is calculated as the sum of number of failures on each day within the year.

### The impacts of climate change on a stationary water system

The impact of climate change is firstly studied by assuming the conditions of the water system (i.e., the size and distribution of the pipe types and ages) and its failure rate remains unchanged in the next 80 years. This assumpition is commonly used to isolate the impacts of climate change^21^.

**Figure 15:**
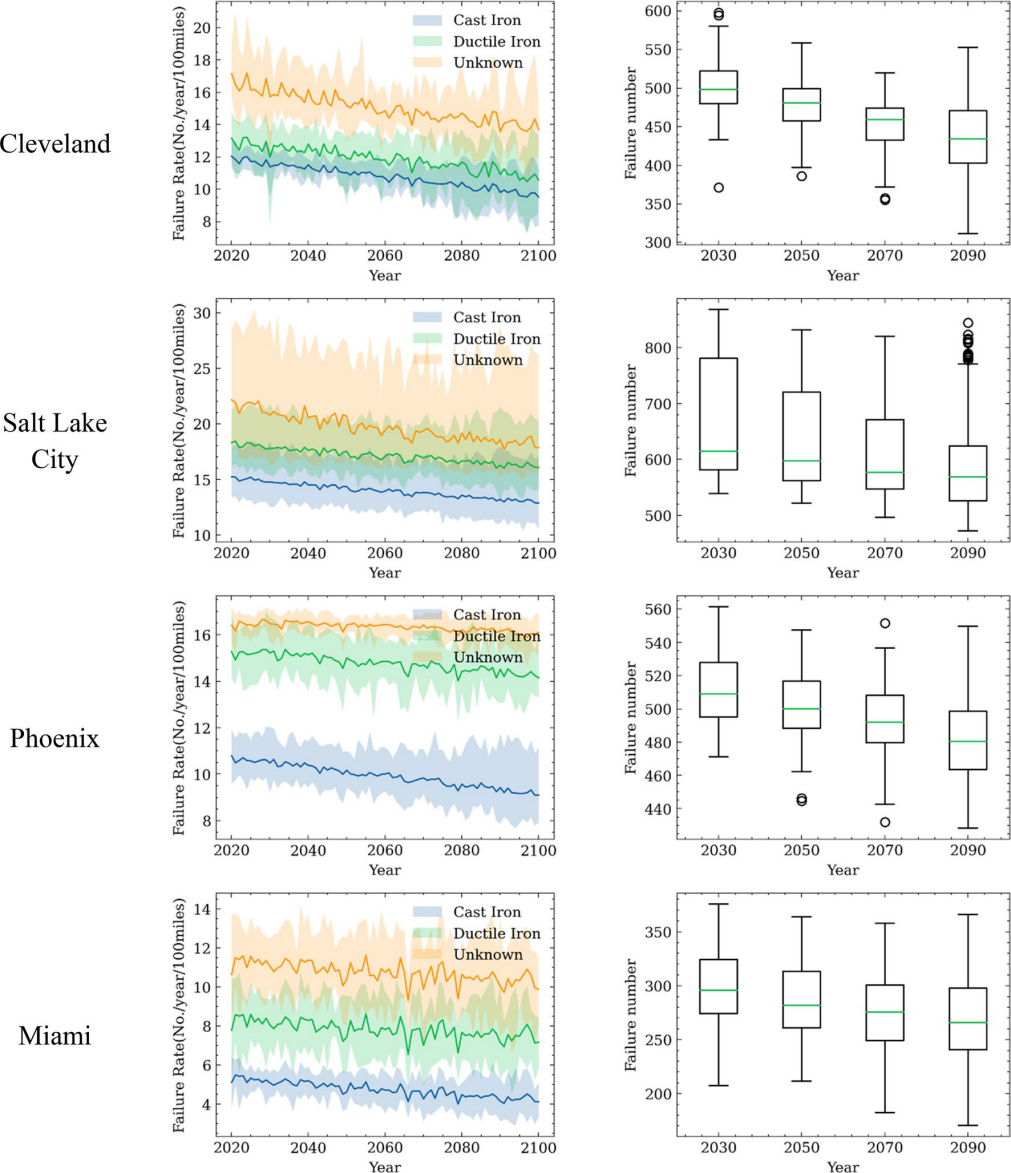
Predicted water system failure for a stationary water system subjected to climate change over 80 years (2020–2100) : left) the failure rates of water pipes in the stationary system made of different materials; right) statistics of total number of failures in the water system grouped into 20-year bins.

Based on this assumption, Fig. 15 shows the simulated water system failures under different climate change scenarios assuming a stationary water system with no aging effects. The left column shows the pipe failure rates of different materials, which are computed by the weighted sum of failure rates of pipes at different age groups, as shown in Eq. (12). For each year, nine failure rates (combination of three climate models and three climate change scenoris corresponding to different socio-economic assumptions) are computed. The results indicate that regardless of the materials of replacement pipe, failure rates of the water system show a decreasing trend in the next 80 years under all climate change scenoris. The cast-iron pipes show a lower failure rates than the ductile iron pipes, and the unknown material pipe has the highest failure rate. Ductile iron pipes show a higher failure rate possibly due to a large number of these pipes are more than 25 years old. A similar trend can also be observed in Fig. 3(b), as over time we can see that the failure rate of ductile iron pipes approaches and then exceeds the failure rate of cast iron pipes.

With the climate fragility obtained from Cleveland Water system data, the same water system located at different geographic locations experience different failure rates. The pipes in regions with lower average temperatures (i.e., Cleveland and Salt Lake City) have higher failure rates than regions with higher average temperatures (i.e., Miami and Pheonix). Moreover, the failure rates of ductile iron pipes are higher than those of the cast iron pipes in high-temperature regions. This is due to that the ductile iron pipes have higher failure rates than cast iron pipes under high temperatures, as shown in Fig. 9(b).

The right column of Fig. 15 shows the distribution of annual failure numbers for the same hypothetic water system located in different cities over each 20-year time span. For each box, the bottom line indicates the first quartile (25%), and the top line indicates the third quartile (75%) of the annual failure numbers. The two extended horizontal lines indicate the 1.5 times interquartile range. Data beyond the range is regarded as outliers. The middle line inside the box is the median value of the distribution of the number of annual pipe failures. A general decreasing trend of the number of water pipe failures can be observed for all the cities in the next 80 years considering the future climate change. This is due to the lower water pipe failure rates at higher temperatures. The results also show that Salt Lake City may experience the largest amount of pipe failures among these cities, probably because of the combined impacts of colder winter and lower precipitation, both increases the water pipe fragility. Miami, FL, where has high temperatures and large precipitation, is predicted to experience the smallest number of failures.

Table 4 summarizes the mean value of the predicted annual number of water pipe failures at different cities over each of the 20-year period during the next 80 years. The results indicate the number of water pipe failures will decrease under warmer weather associated with climate change. For water system in Cleveland, the mean number of water pipe failures decreases from around 500 per year to around 430 per year. This implies that rising temperature due to climate change reduces water system fragility in Cleveland, a potential good news for local water agency. For water system located in Phoneix, the mean number of failures decreases from 598 to 480.

**Table 4:** Median failure numbers of the water system in 20 years period.

Location	2020–2040	2040–2060	2060–2080	2080–2100
Cleveland	498	481	459	434
Salt Lake City	615	597	576	571
Phoenix	508	499	492	480
Miami	295	281	275	265

### The impacts of climate impacts with a dynamic-evolving water system subjected to different replacement strategies

This section explores how the failure rates and numbers change when the water pipes in the water system is subjected to replacement strategies. The pipe age and failure numbers are the two key criteria used to determine water pipe replacement needs^51^. There are also studies related to more advanced pipe replacement strategies^52,53^. In this study, we assumed the pipes’ designed service life is 100 years and minimum service life is 50 years. For each pipe, it will be replaced either when it reaches 100 years old or its annual failure number is larger than 4. The failure number of a pipe is computed from the failure rate determined in "Evolution of pipe failure rate with age for pipes made of different materials" section and its pipe length. We assume this replacement criteria will be used over the next 80 years. Three types of replacement schema are considered, i.e., (1) replace damaged pipes with pipes made of the same material, (2) replace damaged pipes with cast iron pipes, or 3) replace damaged pipes with ductile iron pipes. It should be noted that this is a simplified assumption. In reality, the pipes are replaced based on more complex criteria, such as the break rate trend, pavement protection, fire-fighting flow requirement, water quality, etc.

With the three water pipe replacement schema, the failures of water system in the next 80 years considering different climate change are analyzed. For each year, nine failure rates (combination of three climate models and three climate change scenarios corresponding to different socio-economic assumptions) are computed.

**Figure 16:**
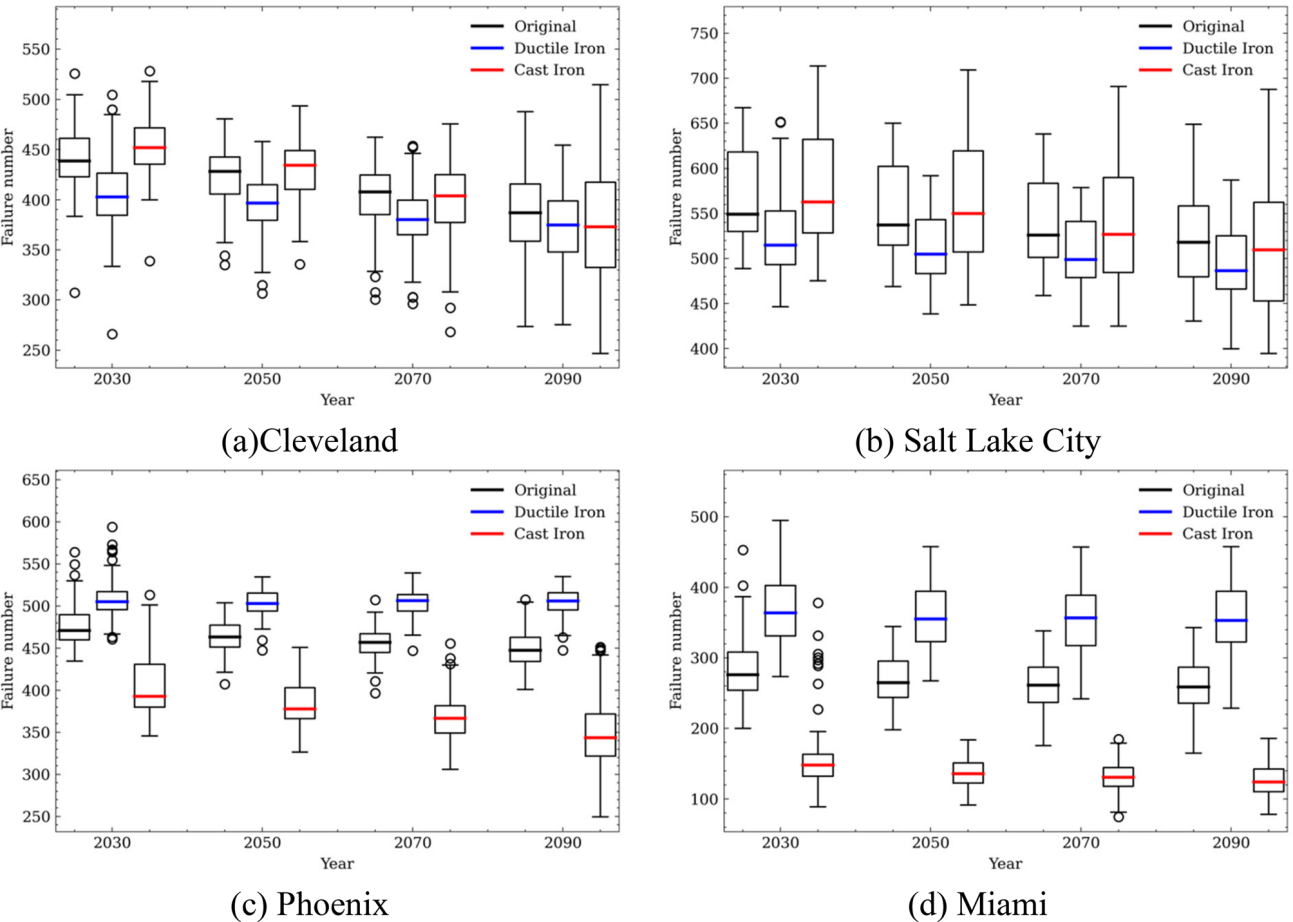
Predicted number of pipe failure in a dynamic water system subjected to different replacement schema under the influence of climate change over 80 years (2020–2100).

Figure 16 shows the statistics of failure numbers over the next 80 years categorized into 20-year bins considering the effects of climate change and three different pipe replacement schema. The annual failure numbers show a more complex trend since the water system changes dynamically due to replacement. The boxes show the distributions of the total failure numbers of every 20 years. The different colors indicates the results of the total water system failure using pipes made of different replacement schema (or replacement with different types of materials).

The results show that for a dynamic water system subjected to different replacement schema, the compound effects of climate changes on water system failure are more complex than only considering the climate change over a stationary water system. In colder regions such as Cleveland and Salt Lake City, the results in Fig. 16 indicate that replacing the failed pipes with ductile iron pipes lead to smallest number of pipe failures in the water system among the three different replacement schema. The reason is possibly that ductile iron pipes are less sensitive to cold temperatures than the cast iron pipes. While for warm regions such as Pheonix and Miami, replacing the failed pipes with cast iron pipes lead to smaller number of pipe failures. This is possibly due to that aged ductile iron pipes have higher failure rates than the cast iron pipes. It is also noticed that replacing the pipes with pipe made of their original materials did not achieve the lowest failure numbers at any time period during the next 80 years.

Overall, the observations indicate for high-temperature regions such as Phoenix and Miami, replacing the old or failed pipes with cast iron pipes achieves a better water system performance; while for low-temperature regions such as Cleveland and Salt Lake City, replacing the old or failed pipes with duct iron pipes achieves a better water system performance.

**Figure 17:**
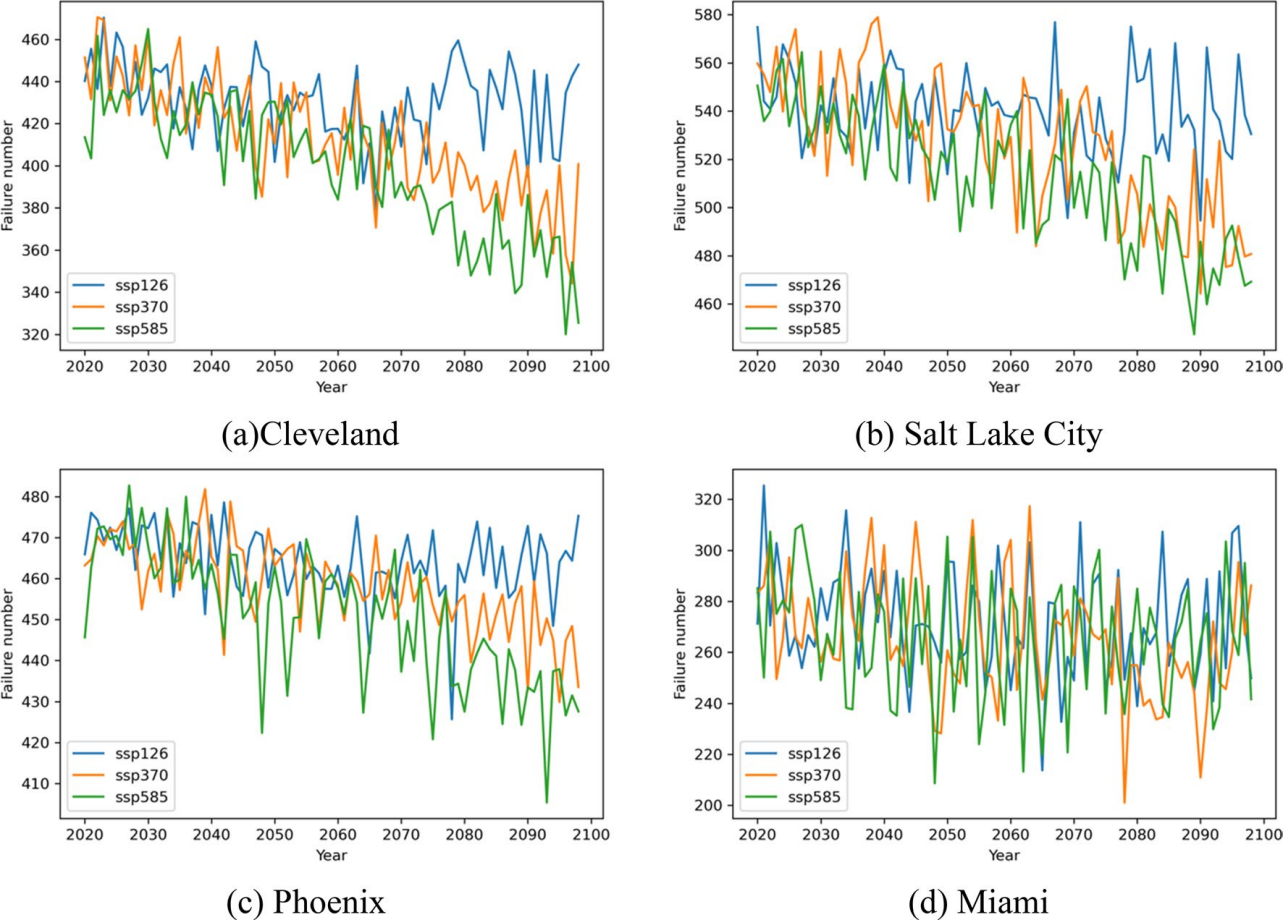
Failure numbers at different shared socioeconomic paths when replacing pipes with original materials.

To break down the impacts of climate change on the water distribution system’s failures at different locations. The mean failure numbers of water system located at each city studied under different climate change scenorios (or different shared socioeconomic paths) are visualized in Fig. 17. The ‘Original’ replacement schema, i.e., the failed pipes are replaced with pipes made of the same materials, is assumed in the analyses. The results indicate that climate change has a significant impact on the overall trends of water pipe failures, especially for water system located in Cleveland, Salt Lake City, and Phoenix, where decreasing trends of pipe failures are predicted. A smaller number of pipes is projected to fail under climate scenarios SSP 585 than those under SSP 126. Comparatively, the climate change impacts are less significant for water system in Miami.

## Conclusions

This study analyzed the climate fragility of water pipes and subsequently extended this to evaluate the impacts of climate change on water system failures. The climate fragility analysis was conducted using data from a large water supply system, which contains water system infrastructure asset consisting of more than 51,832 pipes and 29,621 failure records. The water system database was augmented with climate data. From these, the fragility of water pipes to the two major climate variables, i.e., temperature and precipitation are obtained. Fragility models of water pipes to single climate variable (temperature or precipitation) and combined climate variables (temperature and precipitation) are determined from the historical climate data and pipe maintenance records.

The fragility models showed that the climatic temperature and precipitation have different impacts on pipes depending on their ages and materials. Pipes have a higher failure rate under cold below-freezing temperature, which is consistent with experience by professional engineers. It is also found that old pipes also have higher failure rates under high temperatures, possibly due to higher corrosion rates under such conditions. The precipitation is found to correlate negatively with the pipe failures. Generally, higher precipitations will decrease the pipe failure rates, except for the newly installed cast iron pipes. This is possible because newly installed cast iron pipes are more susceptible to ground movement associated with large precipitation.

Based on the developed failure rate fragility models for both temperature and precipitation, the number of pipe failures in the water supply system can be estimated considering their geographic locations and corresponding climate conditions. The future climate projection at different geographic locations is simulated with different climate models and different climate change scenarios under corresponding social-economic assumptions. Combining the climate fragility model and future climate projection, the influence of climate change on the water supply system is investigated. The influence of water pipe replacement strategies is also evaluated. The results indicate warmer weather associated with climate change overall will decrease the number of water pipe failures. For high-temperature regions, replacing the old or failed pipes with cast iron pipes achieves a better water system performance, while for low-temperature regions, replacing the old or failed pipes with duct iron pipes achieves a better water system performance. Overall, water systems located in the cold regions are projected to experience less number of pipe failures, possibly due to warmer weather associated with climate change.

It is noted that this study only used one water system as the benchmark. The analyses of Cleveland Water data are conducted to determine the fragility of water pipe failure over climate factors (temperature or precipitation). Calibration of climate fragility of water supply system at different geographic regions will help further improve the analyses.

It is also noted that the analyses conducted in this study only considered the fragility due to temperature and precipitation. Climate change-induced natural hazards such as flooding and hurricanes are not considered in the analyses. These hazards can have an important impact on the water supply system, such as water system failure under flood in Jackson, Mississippi, USA.

## Data Availability

The data that support the findings of this study are available from Cleveland Water Division but restrictions apply to the availability of these data, which were used under license for the current study, and so are not publicly available. Data are however available from the authors upon reasonable request and with permission of Cleveland Water Division.
